# Parasites do not adapt to elevated temperature, as evidenced from experimental evolution of a phytoplankton–fungus system

**DOI:** 10.1098/rsbl.2021.0560

**Published:** 2022-02-16

**Authors:** Charlotte Schampera, Ramsy Agha, Florent Manzi, Justyna Wolinska

**Affiliations:** ^1^ Leibniz Institute of Freshwater Ecology and Inland Fisheries (IGB), Berlin, Germany; ^2^ Department of Biology, Chemistry, Pharmacy, Institute of Biology, Freie Universität (FU) Berlin, Berlin, Germany

**Keywords:** adaptation, global warming, host–parasite interaction, disease, cyanobacteria, chytrid

## Abstract

Global warming is predicted to impact the prevalence and severity of infectious diseases. However, empirical data supporting this statement usually stem from experiments in which parasite fitness and disease outcome are measured directly after temperature increase. This might exclude the possibility of parasite adaptation. To incorporate the adaptive response of parasites into predictions of disease severity in a warmer world, we undertook an experimental evolution assay in which a fungal parasite of phytoplankton was maintained at elevated or control temperatures for six months, corresponding to 100–200 parasite generations. Host cultures were maintained at the respective temperatures and provided as substrate, but were not under parasite pressure. A reciprocal infection experiment conducted after six-month serial passages revealed no evidence of parasite adaptation. In fact, parasite fitness at elevated temperatures was inferior in parasite populations reared at elevated temperatures compared with those maintained under control temperature. However, this effect was reversed after parasites were returned to control temperatures for a few (approx. 10) generations. The absence of parasite adaptation to elevated temperatures suggests that, in phytoplankton–fungus systems, disease outcome under global warming will be largely determined by both host and parasite thermal ecology.

## 1. Introduction

Under global warming, the prevalence and severity of some infectious diseases is predicted to change (e.g. [1]). Controlled experiments have shown that elevated temperatures can alter parasite reproduction, infectivity and virulence, and affect host resistance (reviewed in [2–4]). Current knowledge is based mostly on experiments in which host and parasite responses are measured directly after temperature increase, exposing the host to elevated temperatures just for a single generation, and at most a few generations in the case of the parasite (e.g. [5–7]). Such designs exclude potential evolutionary changes that might otherwise lead to thermal adaptation. Additionally, most studies do not test whether observed changes in fitness result from thermal adaptation or phenotypic plasticity. Considering that (micro)parasites can evolve rapidly and that such evolution operates at ecologically relevant timescales (e.g. [8]), adaptation to rising temperatures potentially represents a critically overlooked aspect that might challenge current predictions of disease dynamics under future climate conditions.

The inclusion of organisms' adaptive response to rising temperatures can be addressed by means of experimental evolution: organisms can be maintained over many generations under experimental conditions (i.e. *serial passage experiments*) and their fitness can be evaluated over time, thereby testing for adaptation (e.g. [9,10]). Experimental evolution assays have demonstrated thermal adaptation in non-pathogenic bacteria [11], yeast [12], protozoa [13], green algae [14] and fruit flies [15]. However, thermal adaptation in the context of host–parasite interactions has rarely been investigated.

Phytoplankton is responsible for up to 50% of the global carbon fixation, representing a key driver of biogeochemical cycles and the base of most aquatic food webs [16]. Infection of phytoplankton by fungal parasites (chytrids) can have important ecological consequences for aquatic ecosystems, as it often develops into epidemics capable of severely decimating phytoplankton populations, causing successional changes, and delaying or suppressing algal blooms [17]. Global change favours conditions that promote the dominance of certain harmful phytoplankton groups, e.g. cyanobacteria [18], and blooms of these organisms are indeed expected to increase in frequency and intensity as a consequence of climate change [19]. Given the role of chytrid parasites as strong top-down control agents of phytoplankton populations [17], it is necessary to integrate their response to global warming when projecting phytoplankton dynamics in the future. Although a few experiments have addressed the influence of elevated temperature on the outcome of chytrid infection of phytoplankton [7,20], these studies only reported short-term responses and did not test for adaptation over longer timeframes to sustained elevated temperatures.

To fill this gap, we evaluated the effect of sustained elevated temperature on the fitness of a fungal parasite commonly infecting a widespread freshwater bloom-forming cyanobacterium. We conducted an experimental evolution assay in which parasite populations were maintained under elevated (22°C) and control (16°C) temperatures for six months (100–200 generations) on host populations that were acclimated at the respective temperature but were not under parasite pressure, i.e. did not co-evolve. After this period, the performance of each parasite population was assessed under both elevated and control temperature environments. We expected to detect parasite thermal adaptation, i.e. that parasites reared under elevated temperature would show higher fitness under elevated temperature than their conspecifics maintained under control conditions.

## 2. Material and methods

### (a) Host–parasite system

The monoclonal strain NIVA-Chy-Kol2008 of the chytrid fungus *Rhizophydium megarrhizum* was isolated from a single sporangium from Lake Kolbotnvatnet, Norway in 2008 [21] and used for the experiment. *Rhizophydium megarrhizum* is a lethal obligate parasite (i.e. parasitoid) of phytoplankton, characterized by a motile, free-living life-stage (i.e. zoospores). Chytrid zoospores locate their host by chemotaxis, attach and encyst on the host surface. Chytrids then penetrate the host and form rhizoids to extract nutrients from it, gradually developing into sporangia, reproductive structures that release new haploid zoospores upon maturation [22]. The infection always results in the death of the host. In addition to this asexual mode of reproduction, sexual reproduction has been described for some parasitic chytrid species upon gametangial copulation of resting spores formed under suboptimal growth conditions [22], although it has never been observed in *R. megarrhizum*. Assuming the lack of standing genetic variation in the monoclonal parasite populations used, this limits the potential evolutionary mechanisms in our experiment to mutations, and perhaps marginal inbreeding, which could not be fully ruled out. Although unlikely, it is also possible that the strain has accumulated some genetic variation since its isolation, potentially leading to selection acting on standing genetic variation. Yet, this chytrid strain displayed phenotypic adaptation to novel host environments in a similar timeframe elsewhere [23], demonstrating high adaptive potential despite the likely absence of standing genetic variation.

The host used to maintain the parasite was the filamentous planktonic cyanobacterium *Plankthotrix agardhii*, strain NIVA-CYA630 (isolated from Lake Lyseren, Norway in 2008), a common bloom-forming taxon in temperate lakes. The host was routinely cultivated in Z8 medium [24] as non-axenic batch cultures at 16°C under 20 µmol m^−2^ s^−1^ continuous white-fluorescent light. The parasite was routinely maintained under the same conditions by transferring zoospore suspensions and infected host filaments into uninfected host cultures every three weeks.

### (b) Serial passage experiment

**Figure 1.**
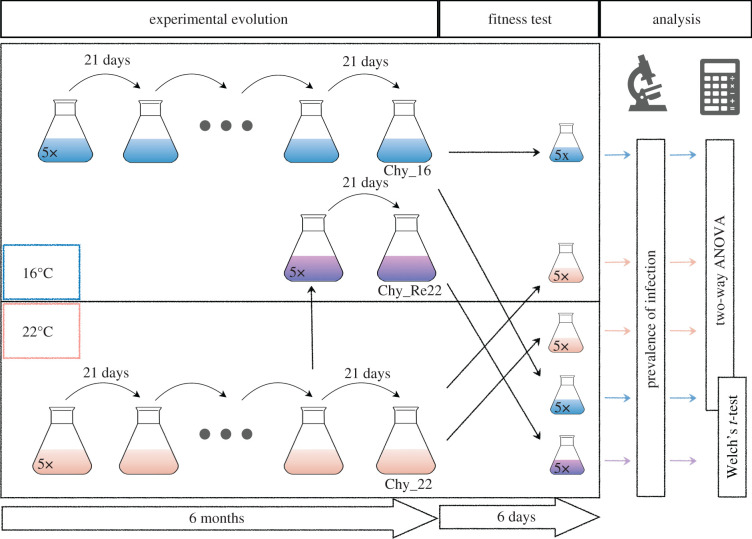
Graphical depiction of the experimental set-up. The chytrid parasite was maintained on its cyanobacterial host at 16°C (control temperature; Chy_16, blue flasks) and 22°C (elevated temperature; Chy_22, red flasks) in five replicates. After a six-month serial passage, parasite fitness was evaluated at control (16°C, upper box) and elevated temperature (22°C, lower box), using the prevalence of infection as a proxy. To evaluate the plasticity of fitness differences, a sub-culture of Chy_22 was transferred to 16°C three weeks before the fitness test (Chy_Re22, purple flasks). The fitness of Chy_Re22 was evaluated at 22°C and compared with Chy_16 at 22°C.

The experimental set-up is depicted in figure 1. Chytrid parasite populations were reared for six months (nine serial passage events, *ca* 100–200 parasite generations) as five independent replicate serial passage lines at either 16 or 22°C (i.e. control and elevated *maintenance temperatures*, respectively), covering the range of the thermal tolerance of the host in temperate lakes [25]. For simplicity, these are referred to as Chy_16 and Chy_22. Hosts provided to the experimental parasite populations were pre-acclimated for one month to the respective maintenance temperature (16 or 22°C) before the onset of the experiment and cultivated under these conditions over the course of the passage experiment as semi-continuous, exponentially growing cultures (optical density at 750 nm (OD_750_) 0.05). Each parasite population was maintained by transferring 3 ml aliquots of the respective infected cultures (containing free-swimming chytrid zoospores (more than 3 × 10^4^) and cyanobacteria with attached parasite sporangia) into 30 ml suspensions of uninfected hosts every three weeks. Resulting high host densities (OD_750_ ≈ 0.05; corresponding to approximately 5000 host filaments ml^−1^) provided non-limiting conditions for ideal parasite transmission.

### (c) Reciprocal fitness test

After the six-month serial passage, parasite fitness tests were conducted at temperatures of 16 and 22°C (*test temperatures*). This resulted in the following design: *2 maintenance temperatures × 5 parasite experimental populations × 2 test temperatures × 5 technical replicates = 100 experimental units*. The fitness tests were conducted in Corning Costar 24-well plates. Each well contained 1.5 ml of exponentially growing uninfected host culture (OD_750_ 0.05), which originated from a separate culture (i.e. not used for the serial passage) which had been pre-acclimated to 19°C (i.e. the mean of the two maintenance temperatures) for two weeks. This was done to avoid putative temperature-specific differences in host physiology that might influence parasite transmission during the fitness tests [26]. Each well was inoculated with zoospores (2000 ml^−1^) obtained from the corresponding experimental parasite population, previously filtered through a 5 µm polycarbonate filter (Whatman Nuclepore Track-Etched membrane). For every resulting pure suspension, the zoospore density was determined using a Sedgewick Rafter counting chamber under a Nikon Ti Eclipse inverted microscope upon fixation of an aliquot in iodine Lugol solution. The plates were incubated at the respective test temperature for 6 days. Well contents were then fixed in 2% paraformaldehyde and stored at 4°C until analysis. The prevalence of infection (i.e. the proportion of infected individuals in a population) was determined by microscopic inspection of 100 random host filaments for the presence of chytrid sporangia and used as a proxy of parasite transmission. Infection prevalence over time follows a sigmoidal curve (e.g. [7]). The prevalence of infection at day six post-inoculation was selected, as it corresponds to the exponential part of such a sigmoidal curve (i.e. highest slope/rate of change), making it a suitable timepoint to detect differences in parasite transmission, which in turn is a commonly used proxy of overall parasite fitness (e.g. [20,23,27]). All sample identities were blinded before the prevalence of infection counts.

### (d) Reversibility fitness test

To test whether potential fitness differences among parasite populations were reversible, an additional test was conducted. Specifically, three weeks before the onset of the fitness test, sub-cultures of Chy_22 were transferred back to the control temperature (16°C) (these populations are referred to as Chy_Re22). In the fitness test, parasite transmission of Chy_Re22 (*5 parasite populations × 5 technical replicates = 25 experimental units*) was then compared with that of Chy_16 at 22°C.

### (e) Statistical analysis

To test for the effects of sustained exposure to elevated temperatures on parasite fitness (as mean infection prevalence of each parasite population), a linear model was fitted, including ‘*maintenance temperature*’ and ‘*test temperature*’ as fixed factors. The proportion of variance explained by each term was calculated as the sum of squares error quotient. To identify differences between the levels of the factors, a contrast test was performed, and *p*-values were corrected for multiple comparisons after Benjamini & Hochberg [28]. To test for reversibility of the observed adaptation, a two-sided Welch's *t*-test was conducted comparing the performance of Chy_16 and Chy_Re22 at 22°C. Model assumptions were confirmed by visual examination of residuals. All statistical analyses were performed in R (v. 3.6.1). Code and data are available as electronic supplementary material.

## 3. Results and discussion

**Figure 2.**
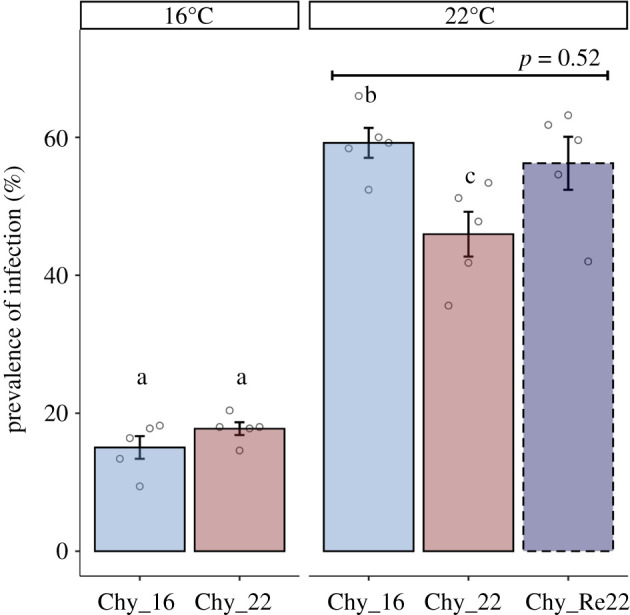
Infection prevalence of parasite populations Chy_16 and Chy_22 (maintained for six months at control (16°C) and elevated temperatures (22°C), respectively), as well as Chy_Re22 (i.e. a subpopulation of Chy_22 returned to 16°C three weeks before the fitness test; dashed-edge bar) at the different temperatures at which the fitness tests were performed (16°C left; 22°C right). Different letters represent significant differences after contrast test and subsequent *p*-value correction (*p* < 0.001). The horizontal bar depicts a two-sided’ Welchs *t*-test comparing Chy_16 and Chy_Re22 at 22°C. Error bars represent s.e.

To investigate the potential for parasite adaptation in a global warming context, we studied the adaptive response of a chytrid parasite to elevated temperatures. *Test temperature* had a stronger effect on parasite transmission than *maintenance temperature* (88.7% of variance explained, table 1). Specifically, regardless of their temperature exposure history, parasites reached threefold higher infection prevalence under the elevated test temperature (22°C) compared with the control temperature (16°C, figure 2). This reflects the eco-physiological effect of temperature on parasite transmission (e.g. [29,30]), reported elsewhere for this host–parasite system [7,20], supporting predictions that the severity of some infectious diseases will increase in a warmer world, in agreement with the metabolic theory [30]. In general, the outcome of host–parasite interactions depends on the interplay between many host and pathogen traits, each with their own thermal performance [31].

**Table 1.** Linear model for the prevalence of infection of parasite populations Chy_16 and Chy_22 (maintained for six months at control (16°C) and elevated temperatures (22°C), respectively, then tested at both temperatures reciprocally) with fixed factors *test temperature*, *maintenance temperature* and their interaction. Significant *p*-values are shown in italics.

factor	d.f.	% explained variance	*F*-value	*p*-value
*test temperature*	1	88.72	278.79	*<0.001*
*maintenance temperature*	1	1.87	5.89	*0.027*
*maintenance temperature* × *test temperature*	1	4.31	13.56	*0.002*
*residuals*	16	5.10		

Besides the thermal eco-physiological response, a six-month serial passage experiment provided no evidence for parasite adaptation to elevated temperatures. At the 16°C *test temperature*, parasite fitness was similar regardless of evolutionary history. Strikingly, at a test temperature of 22°C, parasites maintained at higher temperatures (i.e. Chy_22) showed inferior performance compared with populations kept at control temperature (i.e. Chy_16, figure 2 and table 1). This suggests fitness costs associated with reproduction at sustained higher temperatures and indicates that disease incidence might decrease under higher temperatures in this host–parasite system. To test whether the inferior performance of parasites maintained at higher temperature was reversible, parasites maintained at 22°C were transferred back to 16°C for three weeks (Chy_Re22). Their transmission success at elevated temperatures was then assessed again and compared with that of the parasite lines maintained permanently at 16°C (Chy_16). No difference in prevalence of infection was found between Chy_Re22 and Chy_16 (figure 2), suggesting that the reduction of parasite transmission observed under sustained exposure to higher temperatures might constitute a plastic response. Yet, asserting the mechanisms behind such a reversed response remains challenging. The reversibility of thermal adaptations caused by mutation can most likely be ruled out in such a short time (three weeks represents about 10 parasite generations at 16°C), so that any phenotypic changes observed within this period could be attributed either to phenotypic plasticity (e.g. via temperature-induced loss of epigenetic modifications [32]), or, alternatively, to reversed selection on standing genetic variation upon the emergence of novel genotypes that did not fully reach fixation during the passage period.

The fact that sustained exposure to elevated temperatures led to inferior parasite performance relative to parasites reared at control temperature is inconsistent with the beneficial acclimation hypothesis (BAH, [33]), which posits that organisms acclimated to a particular environment have enhanced fitness in that environment relative to organisms acclimated to other environments. However, empirical evidence against the BAH is available for some organisms (see, e.g. [33–35]) and has been associated with physiological burdens (e.g. production of stress proteins) or effects of chronic stress that elicit fitness costs associated with (thermal) acclimation. We argue that, in our experiment, this effect of reduced parasite fitness is due to a sustained exposure to a suboptimal cyanobacterial host environment. Cyanobacteria produce a number of bioactive oligopeptide metabolites that have been shown to be involved in antiparasitic defence [36]. Given that oligopeptide cellular contents are directly correlated to growth rate [37] and that Chy_22 was maintained on hosts growing at 22°C, a temperature yielding higher cyanobacterial growth rates [38,39], it is reasonable to assume that the exposure to parasite-deterrent compounds was more severe for Chy_22 than for Chy_16 populations during the six-month serial passage. Thereby, relative parasite fitness might be affected by host physiology, resulting in decreased parasite reproductive output or reduced lifespan of infective stages when exploiting hosts growing at higher temperatures.

## 4. Conclusion

We found no evidence for parasite adaptation to elevated temperatures in the phytoplankton–fungus system studied here. This suggests that disease outcome in this system is mainly determined by the thermal ecology of both antagonists. In the interaction between chytrid and cyanobacteria, short-term experiments may hence be adequate and sufficient to predict parasite response to global warming. However, we caution against generalizing these findings to other host–parasite systems. Instead, we call for additional investigations with further host–parasite models using similar experimental approaches to those presented here. Ideally, in addition to evolution via mutations, other mechanisms of evolution should be explored, such as sexual recombination or clonal turnover. The reversibility of the observed phenotypic changes should be investigated via the use of ‘reset’ treatments and the underlying mechanism elucidated. Additionally, the combined effect of temperature and parasite pressure on evolution of host resistance as well as possible changes in host population dynamics and the influence of fluctuating environments remains to be addressed. This is crucial for integrating potential long-term adaptations into predictions of the effect of global warming on host–parasite interactions.

## Data accessibility

All data are accessible as electronic supplementary material. These include raw data in a csv-file and the R code for data visualization and statistical analysis.
